# Spatial *Anopheles arabiensis* (Diptera: Culicidae) insecticide resistance patterns across malaria-endemic regions of Botswana

**DOI:** 10.1186/s12936-020-03487-z

**Published:** 2020-11-19

**Authors:** Mmabaledi Buxton, Ryan J. Wasserman, Casper Nyamukondiwa

**Affiliations:** 1 Department of Biological Sciences and Biotechnology, Botswana International University of Science and Technology, Palapye, Botswana; 2 Department of Zoology and Entomology, Rhodes University, Makhanda, 6140 South Africa

**Keywords:** DDT, Knockdown time, Pyrethroids, Southern africa, Susceptibility bioassays

## Abstract

**Background:**

Since the advent of the Green Revolution, pesticides have played an important role in the global management of invertebrate pests including vector mosquitoes. Despite optimal efficacy, insects often display insensitivity to synthetic insecticides owing to prolonged exposure that may select for resistance development. Such insecticide insensitivity may regress national and regional coordination in mosquito vector management and indeed malaria control. In Botswana, prolonged use of synthetic insecticides against malaria vectors have been practiced without monitoring of targeted mosquito species susceptibility status.

**Methods:**

Here, susceptibility status of a malaria vector (*Anopheles arabiensis*), was assessed against World Health Organization-recommended insecticides, across three malaria endemic districts. Adult virgin female mosquitoes (2–5 days old) emerging from wild-collected larvae were exposed to standardized insecticide-impregnated papers with discriminating doses.

**Results:**

The results showed resistance dynamics were variable in space, presumably as a result of spatial differences in insecticide use across malaria endemic districts and the types of insecticides used in the country. Overall, there was a reduced susceptibility of *An. arabiensis* for the pyrethroid lambda-cyhalothrin and for dichloro diphenyl trichloroethane [DDT], which have similar modes of action and have been used in the country for many years. The Okavango district exhibited the greatest reduction in susceptibility, followed by Ngamiland and then Bobirwa, reflective of national intervention strategy spraying intensities. Vector mosquitoes were, however, highly susceptible to carbamates and organophosphates irrespective of region.

**Conclusions:**

These results provide important findings of vector susceptibility to insecticides recommended for vector control. The results highlight the need to implement insecticide application regimes that more effectively including regionally integrated resistance management strategies for effective malaria control and elimination.

## Background

Over decades, arthropods have been controlled using synthetic pesticides with dramatic reduction on target pest populations and their associated impacts of society [1, 2]. While effective, their prolonged and widespread use has unintentionally resulted in increased prevalence of pesticide resistance, with agricultural and medical implications [3, 4]. Pesticide resistance is typically a result of injudicious pesticide use and mounting pressure on population genetics (e.g. through natural selection and evolution) [5, 6]. Indeed, pesticide resistance is triggered by many genetic, [7] operational [8, 9] as well as biological [10] factors. For instance, selection pressure due to excessive use of insecticides may result in behavioral adaptation of vectors and subsequently, gene mutation expression, leading to temporal and spatial intra-specific heterogeneity [7]. While well-assessed in certain taxa and regions, pesticide resistance is dynamic in space and time and requires continuous evaluation. There are, however, regions where resistance has not been assessed. Furthermore, without empirical evidence for optimal efficacy, synthetic pesticidal active ingredients continue to be used within same localities.

Malaria in humans is an infectious disease, spread by various mosquito species in the *Anopheles* genus, which serve as bridge vector hosts for the *Plasmodium* spp. parasites that cause the disease. Given the role of mosquitoes in malarial transmission dynamics, their management is a crucial component of integrated malaria control strategies [11, 12]. Mitigation of the spread of malaria is typically reliant of vector monitoring and control, which most often involves the use of insecticides [13], although several other complementary approaches are also widely explored [14, 15]. With regards to insecticide resistance, there are four common synthetic insecticides that are capable of conferring resistance to insects; namely the organochlorines, organophosphates, carbamates and pyrethroids [16]. These insecticides are target site specific with organophosphates and carbamates inhibiting activity of the neuro-synaptic enzymes whilst the organochlorines and the pyrethroids target the sodium ion channels [17, 18]. Mosquitoes have shown alterations in their genetics (e.g. acetylcholinesterase genes), with consequent reduction in the binding efficiency with insecticides and hence reduced efficacy [19]. According to Williamson et al*.* [20], organochlorines and pyrethroid resistance emanates from point mutations in the voltage-gated sodium channels resulting in knockdown resistance (KDR). Cross resistance as a consequence, may occur when a resistance mechanism, also confers resistance to another insecticide [21], thus further occurring between pesticides from different chemical classes [22]. Evidence of multiple insecticide resistance in mosquito vectors, including *Anopheles* species, have been reported from many regions [23, 24]. Furthermore, *Anopheles* malaria vectors have also been shown to develop adaptive escape behaviours, through either learning or based on insecticide avoidance and/or repellency, creating further challenges for control and elimination of these vectors and associated infections [25, 26].

Insecticide resistance is a consistently worsening situation in Africa, requiring urgent intervention for effective control of malaria vector species [27, 28]. Malaria is the most prevalent mosquito-borne disease in the sub-Saharan Africa and is carried by various species of Anopheline mosquitoes. Botswana, situated in the warm subtropics of southern Africa is no exception, with malaria cases reported annually (~ 0.01% /1000 population) and even spreading to non-endemic parts of the country [29]. However, regardless of the pronged insecticide use for vector control in Botswana [30], mosquito susceptibility investigations are scant. For many decades lamda-cyhalothrin (pyrethroid) and dichloro diphenyl trichloroethane (DDT; organochlorine) have been the main insecticides used for mitigation against malaria vectors in Botswana [30], although recently (2019) pirimiphos-methyl (organophosphate) was deployed for indoor residual spraying (IRS) use across all malaria endemic districts [13]. Similarly, since the 1940s, DDT has been used in the country for IRS and later complimented by pyrethroids long-lasting insecticide nets (LLINs) and microbial larviciding (*Bacillus thuringiensis* serovar *israelensis*) [13, 31]. However, insecticide susceptibility status of malaria vectors is currently unexplored in Botswana. This is regardless of the country having been using these insecticides for > 70 years [30, 32], a time-scale that will likely have promoted resistance development. This may subsequently regress nationwide or regional planning initiatives on malaria elimination achievement targets by 2023 [33].

As part of a larger project on mosquito control in the region, here we conducted a baseline assessment on *Anopheles arabiensis* insecticide susceptibility for World Health Organization (WHO) recommended and currently used pesticides across three malaria endemic regions in Botswana [34]. *Anopheles arabiensis* is the biggest contributor to malaria in the region [35] and is widely distributed across malaria endemic and even non-endemic parts of the country [36–38]. Specifically, *An. arabiensis* susceptibility status to eleven registered insecticide products was assessed, comprising four classes of pesticides and determined their knockdown times (KDT_50_) and differences in susceptibility patterns across malaria endemic districts. It was hypothesized that (i) locally used insecticides and those with a similar mode of action, had differed efficacy on *An. arabiensis*, with (ii) knockdown times differed across recommended insecticides based on their mode of action and intensity of use, and (iii) that *An. arabiensis* susceptibility status will differ in space owing to differences in insecticide use.

## Methods

### Mosquito collection and maintenance

**Fig. 1:**
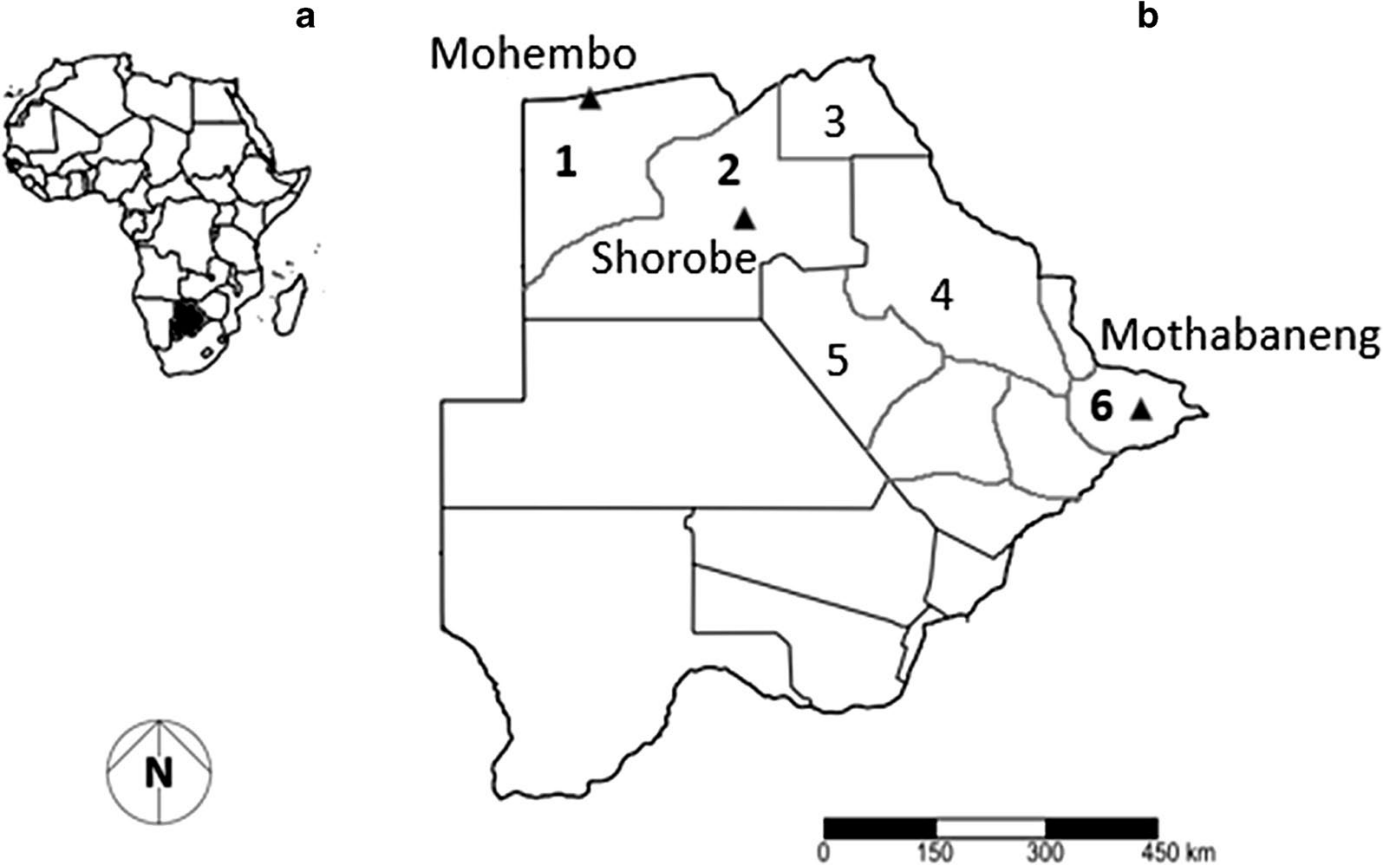
Map showing (**a**) location of Botswana in Africa and (**b**) malaria endemic districts in the northern part of the country: [Okavango (1), Ngamiland (2), Chobe (3), Tutume (4), Boteti (5) and Bobirwa (6)], with study site villages (black up-pointing triangle) in Okavango (Mohembo), Ngamiland (Shorobe) and Bobirwa (Mothabaneng)

Mosquito larvae were collected from stagnant pools across human settlements in malaria endemic districts (Okavango (Mohembo): 18. 2876° S, 021. 7898° E, Ngamiland (Shorobe): 19. 7625° S, 023. 6774° E and Bobirwa (Mothabaneng): 22. 1051° S, 028. 5253° E) in austral summer season between February to March of 2016 and 2017 (Fig. 1). Each sampled district was represented by a village undergoing a deployment of chemical intervention (IRS and/or LLINs) through the country’s national malaria vector control programme. Site selection was based on proximity to human settlements. The collection was done using a 1000 µm mesh net with larvae transferred to a netted 3 L aerated container holding ~ 1.5 L 50:50 habitat and matured tap water, and transported in cooler boxes to the laboratory for further processing. In the laboratory, rearing containers were housed in climate chambers (HPP 260, Memmert GmbH + Co.KG, Germany) set at 25 °C ± 2 and 65% ± 10 relative humidity (RH) under a 12:12 light:dark photocycle. The larvae were fed with fish food (Sera: Vipan family, Randburg, South Africa) ad libitum daily and the water was exchanged with matured tap water every two days to prevent the built up of scum. Eclosed adults were fed with 10% sugar solution ad libitum soaked in a piece of cotton wool placed over the net. Adults were identified using gross morphology [39, 40] and confirmed as *An. arabiensis* following Bass et al*.* [41]. Adult mosquitoes were morphologically sexed upon eclosion using differences in antenna [40] with virgin females retained for use in bioassays.

### Insecticide susceptibility bioassays

The insecticide susceptibility bioassays were performed in accordance with the standard WHO procedure [34]. Virgin female mosquitoes (2 to 5 days old) were exposed (by contact) to surfaces impregnated by a discriminating insecticide doses for a period of 1 h to assess mortality [34]. The bioassays were performed with eleven insecticide types across three districts (*n* = 1100 per sampling site) grouped in four common recommended classes, namely; (i) organophosphates (malathion 5%), (ii) organochlorines (DDT 4.0%, dieldrin 0.4%, dieldrin 4%), (iii) carbamates (propoxur 0.1%, bendiocarb 0.1%) and (iv) pyrethroids (lambda-cyhalothrin 0.05%, permethrin 0.75%, deltamethrin 0.5%, cyfluthrin 0.15% and etofenprox 0.5%). A batch of 20 virgin female mosquitoes were transferred into a total of 5 holding tubes (*n* = 100 per insecticide), four of which (green dotted) were replications to be exposed to insecticides while the other was used as a control. A set of exposure tubes, four lined with impregnated insecticide (red dotted) and a control tube consisted of a paper lined with risella oils (organochlorines), silicone oils (pyrethroids) and olive oils (organophosphates and carbamates). The holding tubes and the exposure tubes were fastened together, and mosquitoes were transferred to the exposure tubes. Mosquitoes that were damaged during the transfer were replaced before the exposure period. After a successful transfer, the holding tubes were removed leaving the exposure tubes upside down on the slides. For every 15 min (up to 60 min), the knockdown mortalities were observed and recorded. At the elapse of 1 h, the mosquitoes were transferred back to the holding tubes then kept for the next 24 h with a cotton wool (soaked in 10% sugar solution) placed on top for feeding purposes. Mosquitoes that survived 24 h post this treatment may have developed resistance [34].

### Data analysis

Mosquito mortality for insecticide susceptibility testing was analysed as percentage following corrected control mortality [34, 42, 43]. The formula was ignored if control mortality was below 5%, but was used only when the control mortality was between 5 and 20%. However, if control mortality was more than 20%, the experiment was discarded. Overall, mosquito efficacy to tested insecticides were defined as susceptible (≥ 98% mortality), suspected resistance (90- 97% mortality) and resistant (< 90% mortality) [29]. The knockdown time 50% (KDT_50_) of total tested female mosquitoes for each insecticide were pooled together and subjected to probit analysis from the Statistical Package for the Social Sciences (SPSS) software (Version 24) [43, 44].

## Results

### Insecticide susceptibility bioassays

Insecticide resistance (< 90% mortality) was recorded in Okavango and Ngamiland mainly for the pyrethroid pesticidal group (Table 1). Okavango showed prominent resistance to pyrethroids; lambda-cyhalothrin 0.05%, permethrin 0.75%, deltamethrin 0.5% and cyfluthrin 0.15% (78.8%, 78.8%, 81.3% and 83.8% mortality respectively) while Ngamiland mosquitoes exhibited resistance to lambda-cyhalothrin 0.05% and permethrin 0.75% (81.3% and 83.8% mortality respectively). Suspected resistance (90–97% mortality) to organochlorines was confirmed in all study sites (districts) (Table 1). Okavango recorded suspected resistance to DDT 4.0%, and dieldrin dosages (0.4 and 4%) at 96%, 93% and 95% mortality respectively. Similarly, Ngamiland exhibited suspected resistance to DDT 4.0%, and dieldrin dosages (0.4 and 4%) at 97.5%, 95% and 96.3% mortality, respectively, while Bobirwa mosquitoes yielded suspected resistance of 97.5% mortality to dieldrin (0.4%). Moreover, dieldrin (0.4%) showed suspected resistance across all districts tested. Suspected resistance was further observed in pyrethroids across districts sampled. Okavango mosquitoes reported suspected resistance of 97.5% mortality to etofenprox (0.5%). Ngamiland mosquitoes displayed suspected resistance to deltamethrin (0.5%), cyfluthrin (0.15%) and etofenprox (0.5%) at 92.5%, 97.5% and 96.3% mortality respectively. Mosquitoes in Bobirwa showed suspected resistance of 95%, 95% and 96.3% mortality to lambda-cyhalothrin (0.05%), permethrin (0.75%) and deltamethrin (0.5%) respectively. Susceptibility (≥ 98% mortality) to organophosphate (malathion (5%) and carbamates; propoxur (0.1%) and bendiocarb (0.1%) was recorded in mosquitoes from all the districts (Table 1). Bobirwa mosquitoes showed susceptibility to organochlorines; DDT (4.0%) and dieldrin (4%) both at 98.8% mortality. Similarly, they yielded susceptibility to pyrethroids; cyfluthrin (0.15%) and etofenprox (0.5%) both at 98.8% mortality.

**Table 1:** A summary of percentage mortality 24 h after a 1-h exposure to different classes of insecticides on field collected F_1_ progeny of *An. arabiensis* (*n* = 100 per insecticide) from Okavango, Ngamiland and Bobirwa districts (*n* = 1100 per sampling site)

Insecticide		District and resistance status		
Class	Name	Okavango	Ngamiland	Bobirwa
Organophosphates	Malathion (5%)	100 (S)	98.8 (S)	100 (S)
Organochlorines	DDT (4.0%)	96 (SR)	97.5 (SR)	98.8 (S)
	Dieldrin (0.4%)	93 (SR)	95 (SR)	97.5 (SR)
	Dieldrin (4%)	95 (SR)	96.3 (SR)	98.8 (S)
Carbamates	Propoxur (0.1%)	98.8 (S)	100 (S)	100 (S)
	Bendiocarb (0.1%)	100 (S)	100 (S)	98.8 (S)
Pyrethroids	Lambda-cyhalothrin (0.05%)	*78.8 (R)*	*81.3 (R)*	95 (SR)
	Permethrin (0.75%)	*78.8 (R)*	*83.8 (R)*	95 (SR)
	Deltamethrin (0.5%)	*81.3 (R)*	92.5 (SR)	96.3 (SR)
	Cyfluthrin (0.15%)	*83.8 (R)*	97.5 (SR)	98.8 (S)
	Etofenprox (0.5%)	97.5 (SR)	96.3 (SR)	98.8 (S)

Letters in the parentheses indicate resistance status of tested mosquitoes (S: susceptible, SR: suspected resistance and R: resistant). All pesticides indicated in italics symbolize cases of insecticide resistance (< 90% mortality)

### Knockdown time (KDT_50_)

**Fig. 2:**
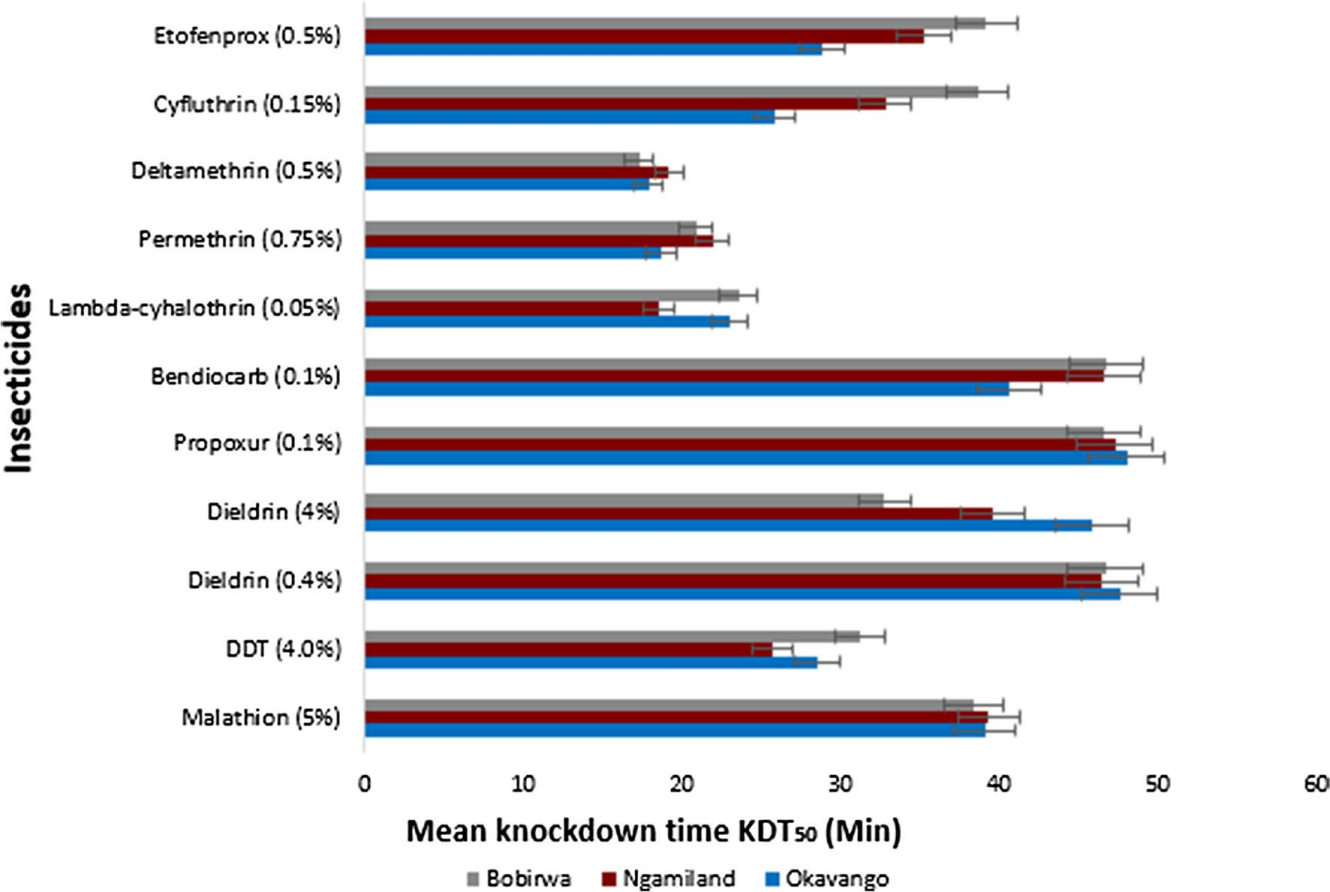
Mean ± 95% CL summary results of knockdown time (KDT_50_) (minutes) of field collected *Anopheles arabiensis* F1 progeny (*n* = 100 per insecticide) from malaria endemic districts tested against different classes of insecticides. Pyrethroids (etofenprox 0.5%, cyfluthrin 0.15%, deltamethrin 0.5%, permethrin 0.75%, lambda-cyhalothrin 0.05%), Carbamates (bendiocarb 0.1%, propoxur 0.1%), Organochlorines (dieldrin 4%, dieldrin 0.4%, dichloro-diphenyl-trichloroethane [DDT]) 4.0%), Organophosphates (malathion 5%)

Carbamates [propoxur (0.1%) and bendiocarb (0.1%)] and organochlorines [dieldrin (0.4 and 4%)] had the highest mosquito KDT_50_ across sampled districts (Fig. 2)_._ The lowest value (32.753 min) was recorded in Bobirwa [dieldrin (4%)] and the highest (47.994 min) in Okavango [propoxur (0.1%)]. DDT 4% was the only organochlorine which had the lowest KDT_50_ with the lowest value (25.721 min) reported in Ngamiland and highest in Bobirwa (31.229 min). Organophosphate [malathion (5%)] showed an intermediate KDT_50_ with consistent values for Okavango (39.073 min), Ngamiland (39.294 min) and Bobirwa (38.352 min). The pyrethroids; lambda-cyhalothrin 0.05%, permethrin 0.75% and deltamethrin 0.5% reported the lowest KDT_50_ compared to other classes of insecticides (Fig. 2). In particular, deltamethrin 0.5% scored the lowest value (17.28 min) in Bobirwa while lambda-cyhalothrin (0.05%) had the highest (23.559 min) for the same district. Conversely, amongst the pyrethroids, cyfluthrin (0.15%) and etofenprox (0.5%) had the highest mosquito KDT_50_ with the highest score (39.137 min) reported in Bobirwa [cyfluthrin (0.15%)] and the lowest (25.798 min) in Okavango [cyfluthrin (0.15%)]. Overall, the locally used insecticides (DDT and lambda-cyhalothrin yielded lower KDT_50_ than carbamates and the organophosphate tested (Fig. 2).

## Discussion

The study highlighted the general reduced efficacy of organochlorines and pyrethroids on *An. arabiensis* across malaria endemic areas. Results showed high resistance development for lambda-cyhalothrin (0.05%), permethrin (0.75%), deltamethrin (0.5%) and cyfluthrin (0.15%) in Okavango and for only lambda-cyhalothrin (0.05%) and permethrin (0.75%) in Ngamiland. It further showed that resistance dynamics were variable in space and likely as a result of differing insecticide intensities and nature of application regimes across districts [31]. In Botswana, a combination of vector control strategy using LLINs (pyrethroid based) and IRS (pyrethroid and DDT) have been practiced continuously over decades with first intervention (DDT) rolled out in Okavango, Ngamiland and Chobe districts in mid-1940s [13, 30]. Okumu and Moore [8] suggested that the two intervention strategies used together, may promote the evolution of insecticide resistance in mosquito populations due to increased pesticide selection pressure. Little information is, however, available regarding extent and difference among national vector control and even domestic insecticide use, across the three districts and its role in resistance development. What is known is that the Okavango and Ngamiland districts are formally considered more problematic for malaria and likely receive more intensive and frequent use of intervention strategies [45].

Botswana has been able to effectively reduce malaria cases over the years through chemical based intervention strategies [13, 31]. Parallel to this achievement, this study demonstrated compromised vector efficacy to insecticides used for IRS and LLINs, similar to observations in other parts of the world [46]. The reduced mosquito sensitivity to insecticides observed in this study may be due to the prolonged (> 70 years) use of IRS (DDT and lambda-cyhalothrin) and massive area-wide distribution of LLINs (pyrethroid impregnated) [32]. Moreover, lambda-cyhalothrin (pyrethroid) and DDT (organochlorine) are insecticides of similar mode of action [47]. Insensitivity to pyrethroids not currently registered for vector control in the country *vis* permethrin, deltamethrin, cyfluthrin and etofenprox was recorded. One of the reasons for this could be the prolonged use of lambda-cyhalothrin [13], with the same mode of action as these pesticides. Okavango showed the most prominent insecticide resistance which may be associated with extensive pesticidal usage since 1940s from the national vector control programme and household interventions, as an area of targeted malaria ‘hotspot’ compared to Ngamiland and Bobirwa [31, 32]. Indeed, the results showed that Bobirwa only had cases of suspected resistance. This may be associated with (i) the vector insecticidal intervention intensification strategy post 2012 in Bobirwa and its categorization as a malaria ‘hotspot’ area [31] and (ii) the interventions are less frequent/intense than other malaria endemic areas (e.g. Okavango and Ngamiland) [48]. Moreover, Simon et al*.* [31] reported public defiance in Bobirwa toward national intervention strategies (e.g. IRS), likely ‘delaying’ *An. arabiensis* resistance in the area. However, the data are only based on samples from one location (village) per district. As such, future work should consider monitoring susceptibility status of malaria vectors in more exhaustive human settlements receiving unique chemical intervention to establish other bio-physical factors contributing to insecticide resistance.

The results showed entire susceptibility to the organophosphate (malathion) and carbamates (propoxur, bendiocarb), irrespective of region. The country’s national vector control strategies are based on insecticides (pyrethroids and DDT) that target one site (voltage-gated sodium channel proteins), which may facilitate selection pressure for possible mutation. Therefore, it may be logical, from the perspective above to use insecticides with different modes of action (e.g. organophosphates and carbamates) on rotational/alternation to improve efficacy while simultaneously managing insecticide resistance [49]. To err on the side of caution regarding the deployment of insecticides in microhabitats (e.g. human habitation structures), abiotic factors shown to influence mosquitocides’ efficacy [50, 51] should also be considered during application. For example, temperature can interact with mosquito chemical intervention approaches (LLINs and IRS) within structures of ‘unstandardized’ thermal condition [50]. As such, assessing temperature coefficient (TC) of pesticides prior to regional use is recommended, as insecticides with positive TC are likely less efficacious at elevated temperatures [51, 52]. Future work on monitoring and evaluation of other *Anopheles* vectors [35] and mechanisms of resistance is warranted (although see Kgoroebutswe et al*.* [38]).

Assessment and selection of pesticides based on their time of action for vector control is an essential component that has a bearing in management of insecticide resistance. The KDT_50_ determines the time that enables 50% of mosquito population to be knocked down by an insecticide. Although it may be necessary to opt for insecticides that are fast in action (shorter KDT), this can be overruled if induced insecticide resistance is observed. The results demonstrated that pyrethroids generally reported shorter KDT_50_ than other classes tested. This is in keeping with Wakeling et al*.* [53] that this group of insecticide is fast in action. Regardless of their ability to knockdown mosquitoes within a short period of time, this group appeared not to be efficacious for *An. arabiensis*, at least in Okavango and Ngamiland, indicating potential development of pyrethroid resistance. In contrast, malathion, propoxur and bendiocarb were generally observed to be slow to action (high KDT_50_) across the study sites with mosquito vector susceptible to their discriminating dozes. Therefore, it may follow that, if mosquitoes do not show resistance, a fast-acting insecticide may be given priority of choice but with pyrethroid-resistance areas (as in Okavango), slow-acting insecticides (e.g. organophosphates and carbamates) may serve as alternatives. Hence, it may be important that vector response assessment on both the KDT_50_ and insecticide susceptibility status be carefully considered and merged appropriately for future insecticide selection and subsequently managing resistance. Furthermore, alternative vector control strategies [54], applied in an integrated and area-wide approach may help bridge resistance development.

Though pyrethroids were observed to be fast in action, *An. arabiensis* displayed a compromised sensitivity to the insecticides, which has implications for future vector control strategies using this pesticidal group. This baseline assessment work advocates for continuous monitoring of insecticide resistance to all potential mosquito vectors in the country before conclusive recommendations on susceptibility status are made. Moreover, malaria elimination in the country and region is a priority that necessitates efforts in vector management to monitor insecticide resistance. This may be achieved using integrated approaches that complement the current vector management strategies in minimizing resistance and at the same time delivering environmental benefits [55].

## Conclusion

While requiring further investigation, the results suggest that *An. arabiensis*, one of the important malaria vectors in the country [35], may be showing a genetic drift towards resistance as reported in other southern African neighbouring countries [56, 57]. The current study adds to other reports on insecticide resistance in Africa and may be extended to other disease vectors on fine-to large-scale susceptibility to insecticides for both the endemic and non-endemic districts across diverse landscapes [43, 58]. With the resistance reported here, Botswana should integrate the current national intervention strategies with other approaches of vector management in minimizing resistance and simultaneously considering environmental benefits. This may include novel complimentary non-chemical ‘bio-friendly’ approaches targeting both immature and adult vector life stages [59, 60]. Furthermore, public records of governmental, industrial and private insecticidal use and availability should be considered to aid delimit drivers of resistance development. This may help integrated vector management, frameworks for pest decision-making, continued insecticide resistance monitoring, and the implementation of insecticide resistance management strategies while maintaining biodiversity and essential ecosystem services [61]. The country needs to be more conservative with the continuous use of pyrethroids especially in the Okavango delta, where insecticide resistance was evident and biodiversity sustenance is key for sustainable livelihoods and the tourism economy [62].

## Data Availability

The datasets during and/or analysed during the current study available from the corresponding author on reasonable request.
